# Viral RNA Levels and *env* Variants in Semen and Tissues of Mature Male Rhesus Macaques Infected with SIV by Penile Inoculation

**DOI:** 10.1371/journal.pone.0076367

**Published:** 2013-10-11

**Authors:** Francis Fieni, Mars Stone, Zhong-Min Ma, Joseph Dutra, Linda Fritts, Christopher J. Miller

**Affiliations:** 1 Center for Comparative Medicine, University of California Davis, Davis, California, United States of America; 2 California National Primate Research Center, University of California Davis, Davis, California, United States of America; 3 Department of Pathology, Microbiology and Immunology, School of Veterinary Medicine, University of California Davis, Davis, California, United States of America; 4 Division of Infectious Diseases, School of Medicine, University of California Davis, Davis, California, United States of America; Emory University School of Medicine, United States of America

## Abstract

HIV is shed in semen but the anatomic site of virus entry into the genital secretions is unknown. We determined viral RNA (vRNA) levels and the envelope gene sequence in the SIVmac 251 viral populations in the genital tract and semen of 5 adult male rhesus monkeys (*Macaca mulatta*) that were infected after experimental penile SIV infection. Paired blood and semen samples were collected from 1–9 weeks after infection and the monkeys were necropsied eleven weeks after infection. The axillary lymph nodes, testes, epididymis, prostate, and seminal vesicles were collected and vRNA levels and single-genome analysis of the SIVmac251 *env* variants was performed. At the time of semen collection, blood vRNA levels were between 3.09 and 7.85 log10 vRNA copies/ml plasma. SIV RNA was found in the axillary lymph nodes of all five monkeys and in 3 of 5 monkeys, all tissues examined were vRNA positive. In these 3 monkeys, vRNA levels (log10 SIVgag copies/ug of total tissue RNA) in the axillary lymph node (6.48±0.50) were significantly higher than in the genital tract tissues: testis (3.67±2.16; p<0.05), epididymis (3.08±1.19; p<0.0001), prostate (3.36±1.30; p<0.01), and seminal vesicle (2.67±1.50; p<0.0001). Comparison of the SIVmac251 *env* viral populations in blood plasma, systemic lymph node, and genital tract tissues was performed in two of the macaques. Visual inspection of the Neighbor-Joining phylograms revealed that in both animals, all the sequences were generally distributed evenly among all tissue compartments. Importantly, viral populations in the genital tissues were not distinct from those in the systemic tissues. Our findings demonstrate striking similarity in the viral populations in the blood and male genital tract tissues within 3 months of penile SIV transmission.

## Introduction

Human immunodeficiency virus (HIV) is shed in semen and disseminated through sexual contact. The virology of HIV in semen has been well studied, particularly in the chronic phase of infection, however little information is available about the anatomic origin of HIV in semen. HIV is present in semen as free viral particles and as infected cells. Some studies have demonstrated that the HIV variants present in semen are distinct from the variants in the blood or other anatomical compartments [1], [2]. Thus, the male genital tissues (MGT) may constitute a compartment that is distinct from the blood and that the viral populations in these sites may be under are under unique selective pressures [3]. Phylogenetic analyses has also shown that cell-free virus variants in the seminal fluid are, in some cases, distinct from those in the infected leukocytes present in the semen [4], [5]. This could be due to passive diffusion of plasma and associated HIV particles into seminal fluid.

SIV infection of macaques is a very useful animal model for studying HIV infection *in vivo* [6], [7]. After intravenous SIV inoculation of male cynomolgous monkeys, virus levels in genital tract tissues are higher during the acute infection than during the chronic infection and the e*nv* variants in the reproductive tract and blood are genetically similar [8]. Despite these initial findings, the extent to which the MGT is a site of virus replication that is capable of generating novel variants remains unclear. The goal of this study was to determine viral RNA (vRNA) levels and the nature of the envelope gene sequence variants in SIV_mac_ 251 viral populations of the genital tract and semen of rhesus macaques that were infected after experimental penile infection.

## Materials and Methods

### Ethics Statement and Animals

The captive-bred 4–9 year old male rhesus macaques (Macaca mulatta) used in this study were from the California Regional Primate Research Center and they were housed in accordance with the recommendations of the Association for Assessment and Accreditation of Laboratory Animal Care International Standards and with the recommendations in the Guide for the Care and Use of Laboratory Animals of the National Institutes of Health. The Institutional Animal Use and Care Committee of the University of California, Davis, approved these experiments (Protocol # 15835). When immobilization was necessary, the animals were injected intramuscularly with 10 mg/kg of Ketamine HCl (Parke-Davis, Morris Plains N.J.). All efforts were made to minimize suffering. Details of animal welfare and steps taken to ameliorate suffering were in accordance with the recommendations of the Weatherall report, “The use of non-human primates in research”. Animals were housed in an air-conditioned facility with an ambient temperature of 21–25°C, a relative humidity of 40%–60% and a 12 h light/dark cycle. Animals were individually housed in suspended stainless steel wire-bottomed cages and provided with a commercial primate diet. Fresh fruit was provided once daily and water was freely available at all times. A variety of environmental enrichment strategies were employed including housing of animals in pairs, providing toys to manipulate and playing entertainment videos in the animal rooms. In addition, the animals were observed twice daily and any signs of disease or discomfort were reported to the veterinary staff for evaluation. For sample collection, animals were anesthetized with 10 mg/kg ketamine hydrochloride (Park-Davis, Morris Plains, NJ, USA) or 0.7 mg/kg tiletamine HCl and zolazepan (Telazol, Fort Dodge Animal Health, Fort Dodge, IA) injected intramuscularly. The animals were sacrificed by IV administration of barbiturates prior to the onset of any clinical signs of disease.

### Penile SIVmac251 Inoculation

The animals used in this study were part of a SIV vaccine study [9] but these animals were naïve controls that were never immunized prior to SIV inoculation. The cell-free SIVmac251 (UCD-6/04) stock used for this study was prepared as described previously [10], [11]. This stock contains about 10^9^ vRNA copies/ml and 10^5^ tissue culture infectious doses (TCID_50_) per ml using CEMX174 cells for tittering. The penis was exposed to virus as described [10] with the following modifications: after the glans and foreskin were exposed to virus for 5 min. the animal was placed back into its cage in dorsal recumbency, and an additional 250 µl of virus was placed in the sulcus between the foreskin and glans using a 1 ml needless syringe.

### Blood Collection and RNA Isolation

Whole blood samples (8 ml) were taken by peripheral venipuncture into anticoagulant (EDTA) weekly and just after semen collection. The blood was centrifuged at 500×g for 10 minutes. The supernatant, corresponding to the plasma, was removed and stored at −80°C awaiting viral RNA extraction. The blood samples were thawed at room temperature and RNA was isolated using the QIAamp Ultrasens Viral Kit (QIAGEN Inc., Valencia, CA) according to the manufacturer’s protocol and eluted in 50 µl.

### Semen Collection, Processing and RNA Isolation

The monkeys were sedated for semen collection; they were placed in a chair and restrained with a leather strap [12]. The animals were monitored continuously for safe positioning, depth of anesthesia, oxygen saturation, and heart rate. To collect the semen, the penis was gently extruded from the preputial sheath; one gel strip was wrapped around the base of the penis and a second just below the tip. The gel strips were connected to a negative and positive electrode, for the base and tip respectively, from defibrillator pads (CON MED Corp, Utica, NY, USA). Pulsations of 70 volts at a rate of 18 pulses/second for 20 milliseconds over a period of 15 seconds were used. A maximum of 4 separate attempts were made. Ejaculates were collected into a 50 ml Falcon tube containing 2 ml of PBS. Samples were allowed to liquefy for 30 min at 37°C before processing; 10 µl were then diluted at 1/100 (v/v) in NaCl solution (4%) and used for hemocytometer counts (Hausser Scientific, Horsham, PA, USA).

The semen was separated into two fractions by centrifugation at 800×g for 10 minutes: seminal plasma (SP) and sperm cells (SC). The supernatant, corresponding to the SP, was removed and divided in two 1 ml cryotubes and stored at −80°C to await viral RNA extraction. The SC pellet was washed by centrifuging three times in PBS at 800×g for 10 minutes. The cell pellets from the third wash were diluted in 3 ml of fetal bovine serum (Gemini BioProducts, Sacramento CA) supplemented with 10% DMSO (DMSO; Sigma-Aldrich, Saint Louis MO). Cells in solution were placed into 3 cryotubes. Cryotubes were stored at −80°C, awaiting subsequent nucleic acid extraction.

To isolate RNA the seminal plasma samples were thawed at room temperature. If necessary, the seminal plasma volume was completed to 1 ml by adding PBS. Then 3 ml of Trizol LS (Invitrogen, Carlsbad CA) was added, vortexed, and incubated for 5 min at room temperature; 400 µl of BCP – phase separation reagent (Molecular Research Center, Inc. Cincinnati, OH) was added, vortexed, and incubated for 5 min at room temperature. Samples were centrifuged at 12,000×g for 10 min at 4°C. The supernatant was carefully recovered and mixed with the same volume of 70% ethanol. Then RNA was isolated using Qiagen RNeasy MinElute columns (QIAGEN Inc., Valencia, CA) according to the manufacturer’s protocol, and eluted in 25 µl.

To isolate RNA the sperm cell samples were thawed at room temperature and centrifuged at 3,000×g for 5 min at 4°C; the supernatant was then carefully removed. The cell pellets were re-suspended in 350 ml of RTL buffer plus (Qiagen kit) and processed to homogenize cell lysates in a QIA shredder spin column (QIAGEN Inc., Valencia, CA). The lysates were then placed in a Qiagen AllPrep DNA/RNA column. The DNA was isolated according to the manufacturer’s protocol, and eluted in 70 µl. The first flow-through from the AllPrep DNA/RNA column containing the RNA was recovered and mixed with the same volume of 70% ethanol. Then RNA was isolated using the Qiagen RNeasy MinElute columns (QIAGEN Inc., Valencia, CA) according to the manufacturer’s protocol, eluted in 25 µl and stored at −80°C, awaiting PCR analysis.

### Tissue Collection and RNA Preparation

Genital tract tissues (testis, epididymis, prostate, and seminal vesicle) and genital lymph nodes (inguinal and iliac lymph nodes) systemic lymphoid tissues (axillary lymph nodes and spleen) and blood were collected at the time of necropsy and analyzed for vRNA levels. Tissues were stored in RNAlater (Ambion, Austin, TX) and kept at −20°C until preparation of RNA. Tissue samples were homogenized with a Power Homogenizer (Power-Gen, Fisher Scientific) according to the manufacturers protocol. Total RNA was isolated using Trizol (Invitrogen, Carlsbad, CA) following the manufacturers suggested protocol. RNA samples were DNase treated with DNA-free (Ambion) for 1 hr at 37°C. cDNA was prepared using random hexamer primers (Amersham-Pharmacia Biotech, Inc.) and SuperScript III Reverse Transcriptase (RT) (Invitrogen, Carlsbad, CA).

### cDNA Synthesis

The RNA isolated from the samples was reverse transcribed into cDNA using SuperscriptIII reagents (Invitrogen, Carlsbad, CA) with 2 µl of 50 µM dT23VN (or gene specific primer for SGA analysis: SIVEnvR1 5′-TGTAATAAATCCCTTCCAGTCCCCCC-3′), 2 µl of 10 mM dNTPs, and 22 µl of viral RNA. This mixture was heated to 65°C for 5 minutes then incubated on ice for 2 minutes. A master mix of the following was then added: 8 µl of 5X First-Strand Buffer, 2 µl of 0.1 MDTT, 2 µl of RNaseOUT™ Recombinant RNase Inhibitor (40 units/µl), and 2 µl of SuperScriptIII RT (200 units/µl), and Incubated at 25°C for 5 minutes, 50°C for 60 minutes, and 70°C for 15 minutes. Subsequently, 1 µl of E. coli RNaseH (5 U/µ) was added and incubated at 37°C for 20 minutes. The cDNA was stored at −80°C until amplification.

### Quantitative SIV RNA Analysis

A well-described RT-PCR was used to detect and quantify SIV*gag* RNA levels in plasma samples [13], [14]. The copy number of SIV*gag* was calculated based on standard curves for a SIV*gag* plasmid spanning a concentration range from 0.1 to 10^8^ copies. Although amplification from wells with 0.1 copies of SIV*gag* was inconsistent, the assays consistently detected SIV *gag* in wells containing ≥1 copy of the plasmid. The results were analyzed with SDS 7900 system software version 2.3 (Applied Biosystems, Foster City, CA, USA). The results for each sample are reported as log10 vRNA copies per ml of plasma RNA. In a series of previous studies [13], [14] we determined that 125 SIV*gag* RNA copies/ml plasma (equivalent to 2.1 log10 SIV*gag* copies/ml plasma) was a conservative cutoff for determining if a sample from a SIV-inoculated monkey was positive.

### Quantitative SIV DNA Analysis

Samples were tested in replicates of 4 reactions carried out in 96-well optical plates (Applied Biosystems, Foster City, CA, USA, Foster City, CA) in a 25 µl reaction volume containing 5 µl DNA and 20 µl Mastermix (Applied Biosystems, Foster City, CA, USA) using the ABI 7900 robotic thermocycler. All sequences were amplified for 2 min at 50°C and 10 min at 95°C followed by 50 cycles of 15 s at 95°C and 1 min at 60°C. The following primer pairs and probes were used: SIVgag forward primer 1, 5′-3′ GGG AGA TGG GCG TGA GAA A, reverse primer, CGT TGG GTC GTA GCC TAA TTT T and probe TCATCT GCT TTC TTC CCT GAC AAG ACG GA. The copy number of SIVgag was calculated using standard curves for a viral gene plasmid spanning a concentration range from 0.1 to 108 copies. To evaluate the specificity and determine the background of the PCR assay, RNA isolated from plasma taken from 11 rhesus macaques that had never been exposed to SIV were subjected to amplification. There was no amplification of SIVgag from any of these plasma samples. Thus, 125 SIVgag copies/ml plasma (equals 2.1 log10 SIVgag copies/ml of plasma) was used as the cutoff for determining whether a sample from a SIV-inoculated monkey was positive. The results were analyzed with SDS 7900 system software version 2.3 (Applied Biosystems, Foster City, CA, USA). The results for each sample are reported as log10 vDNA.

### Single-genome Nested Amplification of SIVmac251 *env*

Near-full-length 2.2-kb SIVmac251 *env* amplicons spanning nucleotides (nt) 197 to 2429 of gp160 were obtained from cDNA of blood plasma, seminal plasma, or tissue vRNA using nested PCR single-genome amplification as preciously described [11]. A 5-fold dilution series was made from cDNA in TE buffer (10 mM Tris [pH 8.0], 0.1 mM EDTA; Integrated DNA Technologies), and nested PCR was performed. The following master mix was made at room temperature: 5 ul of 5# Phusion HF buffer (New England BioLabs, Ipswich, MA), 0.5 ul of 10 mM dNTPs, 0.75 ul of 10 uM 251envF1 (5%-CAG TCT TTT ATG GTG TAC CAG CTT GGA GGA ATG-3%), 0.75 ul of 10 uM 251envR1 (5%-GAG GAT CCA TCT TCC ACC TCT CCT AAG AGT C-3%), 0.2 ul of Phusion Hot Start high-fidelity DNA polymerase (New England BioLabs, Ipswich, MA), and 14.8 ul of double-distilled water (ddH2O). A 23 ul volume of master mix and 2 ul of cDNA or DNA from 5-fold serial dilutions in TE were added to 0.2-ml tubes. The following cycling conditions produced the first-round 2,519-bp PCR product: 98°C for 45 s, 35 cycles of 98°C for 15 s and 72°C for 1.5 min, with a 4°C dwell. Second-round amplification entailed a master mix of 10 ul of 5# Phusion HF buffer, 1 ul of 10 mM dNTPs, 1.5 ul of 10 uM 251envF2 (5%-GGA ACA ACT CAG TGC CTA CCA GAT AAT GGT G-3%), 1.5 ul of 10 uM 251envR2 (5%-GTA GGT CAG TTC AGT CCT GAG GAC TTC TCG-3%), 0.4 ul Phusion Hot Start high-fidelity DNA polymerase, and 33.6 ul of ddH2O. A 48-ul aliquot of this master mix and 2 ul of first-round product were added to 0.2-ml tubes and cycled under the following conditions to produce a 2,316-bp PCR product: 98°C for 45 s, 35 cycles at 98°C for 15 s and 72°C for 1.5 min, with a 4°C dwell. All PCR products were visualized on a 2.0% E-gel precast agarose gel (Invitrogen). The endpoint of dilution was determined to be between the last dilution reaction mixture to show a PCR positive band on the gel and the next dilution. Replicates of 24 PCR mixtures of the last dilution to show a band and of 24 PCR mixtures one dilution below that point were performed. A positive reaction rate of 30% or lower ensured that amplicons were derived from a single template. Replicates were repeated until sufficient PCR-positive reactions were produced. PCR products were purified using a QIAquick 96 PCR purification kit (Qiagen). Amplicons were eluted in 50 ul EB, and both strands were sequenced by Sequetech (Mountain View, CA) as partially overlapping fragments using BigDye Terminator methodologies on an ABI 3730xl DNA analyzer platform. To confirm PCR amplification from a single template, chromatograms were manually examined for multiple peaks, indicating the presence of amplicons that resulted from PCR-generated recombination events, *Taq* polymerase errors, or multiple variant templates, and thus we could ensure proportional representation of individual *env* sequences circulating *in vivo*. Any sequences containing ambiguous nucleotides were not included in the analysis.

### Sequence Analysis

Sequences were aligned using ClustalW [15] and hand edited using Jalview [16] to improve alignment quality. All trees were constructed with Phylip [17] using the neighbor-joining method [18] with Kimura two-parameter distance matrix [19] and bootstrapped for reliability. Sequences with large deletions were omitted from analysis. Within-subject env diversity was analyzed in three ways, as described in detail previously [20], and fell into two distinct levels classified as either “homogeneous” or “heterogeneous”. Briefly, diversity was determined by visually inspecting sequences by using neighbor-joining phylogenies and the Highlighter tool (www.hiv.lanl.gov). Also, distribution of pairwise Hamming distances (HD) within each sample were examined for peak modality; single peaks indicating infection from a single viral variant and multiple peaks reflecting infection arising from viral variants of polyphyletic lineage. Lastly, mathematical modeling was used to test predictions of expected maximum HD against assumptions of infection variant phylogenies. All sequences were deposited in GenBank with accession numbers KF523409–KF523725.

### Statistical Analysis

All calculations and analysis were performed using Prism 5.0a software (GraphPad) on an Apple Macintosh computer. A *Chi* ^2^ test was used to compare the incidence rate of SIV viral RNA in the two fractions of the semen. A student t test was used to compare average SIV RNA concentration in the tissues. To test if the vRNA levels in MGT tissues correlated with plasma vRNA levels, a Spearman’s Correlation test was used. Values of *P*<0.05 were considered to be significant for all tests and all tests were two-tailed.

## Results

### Outcome of Penile SIV Inoculations

As previously reported [24] one of the monkeys (36338) infected after 9 inoculations at 10^3^ TCID_50_; one monkey (36658) became infected after 10 inoculations at 10^3^ TCID_50_ and 8 inoculations at 10^3^ TCID_50_; and three monkeys (37079, 36199, 36589) became infected after 10 inoculations at 10^3^ TCID_50_, 10 inoculations at 10^4^ TCID_50,_ and 2 inoculations in week 20, morning and afternoon at 10^5^ TCID_50_.

### SIV RNA Levels in Blood and Semen

It was possible to collect at least 1 semen sample from each of the 5 SIV positive monkeys between 5 weeks post infection (PI) and necropsy, and a total of 14 samples were collected (Table 1). Blood plasma SIV RNA levels were between 3.09 and 7.85 log10 vRNA copies/ml at the time of semen collection. Of the fourteen semen samples collected, eight samples were SIV RNA^+^ in seminal plasma fraction and 6 were SIV RNA negative. Three of the semen samples that were SIV RNA^+ in^ seminal plasma also had SIV RNA seminal cells (Table 1).

**Table 1:** SIV RNA levels in blood plasma, seminal plasma and semen cells.

Monkey	Time post challenge (week)	Time post Peak (week)	Blood plasma vRNA^a^	Seminal plasma vRNA^a^	Semen cells vRNA^b^
36338	11	8	6.31	positive	negative
36658	6	1	5.10	positive	negative
	11	5	4.09	No semen	No semen
37079	5	1	5.00	3.59	negative
	6	2	4.78	2.50	negative
	9	5	3.31	negative	negative
	11	7	3.09	negative	negative
36199	5	3	7.05	5.93	3.86
	8	6	7.17	5.45	2.55
	9	7	7.60	4.91	3.30
	11	9	7.85	4.94	negative
36589	6	4	5.42	negative	negative
	8	6	5.02	negative	negative
	9	7	4.21	negative	negative
	11	9	5.15	negative	negative

a log10 vRNA copies per ml blood plasma and seminal plasma.

b log10 vRNA copies per ug cellular RNA.

### SIV RNA Levels in Tissues

SIV RNA was present in the axillary lymph nodes of all five infected monkeys but the genital tract tissues were SIV RNA^+^ in only three monkeys (36338, 36199, 36589) (Table 2). Of note, the vRNA levels in the axillary lymph node (6.48±0.50 copies/ug tissue RNA) were significantly higher than in genital tract tissues: testis (3.67±2.16 copies/ug tissue RNA; p<0.05), epididymis (3.08±1.19 copies/ug tissue RNA; p<0.0001), prostate (3.36±1.30 copies/ug tissue RNA; p<0.01), and seminal vesicle (2.67±1.50 copies/ug tissue RNA; p<0.0001). Genital tract tissues from monkeys 36658 and 37079 were negative, although blood plasma samples were positive in both, and semen plasma was positive for monkey 37079. Further, the vRNA levels in all 4 MGT tissues tested (prostate, seminal vesicle, epididymis, testis) directly correlate with plasma vRNA levels (p = 0.0167; r = 0.9747, Spearman’s Correlation) suggesting that the plasma is the major site of vRNA in all the MGT tissues of these animals. Further, the above results suggest that the MGT is not a major site of SIV replication.

**Table 2:** SIV RNA levels in genital tract tissues at 11 weeks post infection.

Monkey	Axillary LN vRNA^b^	Testis vRNA^b^	Epididymis vRNA^b^	Prostate vRNA^b^	Seminal Vesicle vRNA^b^
36338	6.26	2.73	3.13	3.34	2.27
36658	4.88	negative	negative	negative	negative
37079	3.77	negative	negative	negative	negative
36199	7.05	6.14	4.24	4.66	4.33
36589	6.12	2.15	1.87	2.07	1.42

b log10 vRNA copies per ug tissue RNA.

### SIV *env variants* in Tissues

**Figure 1:**
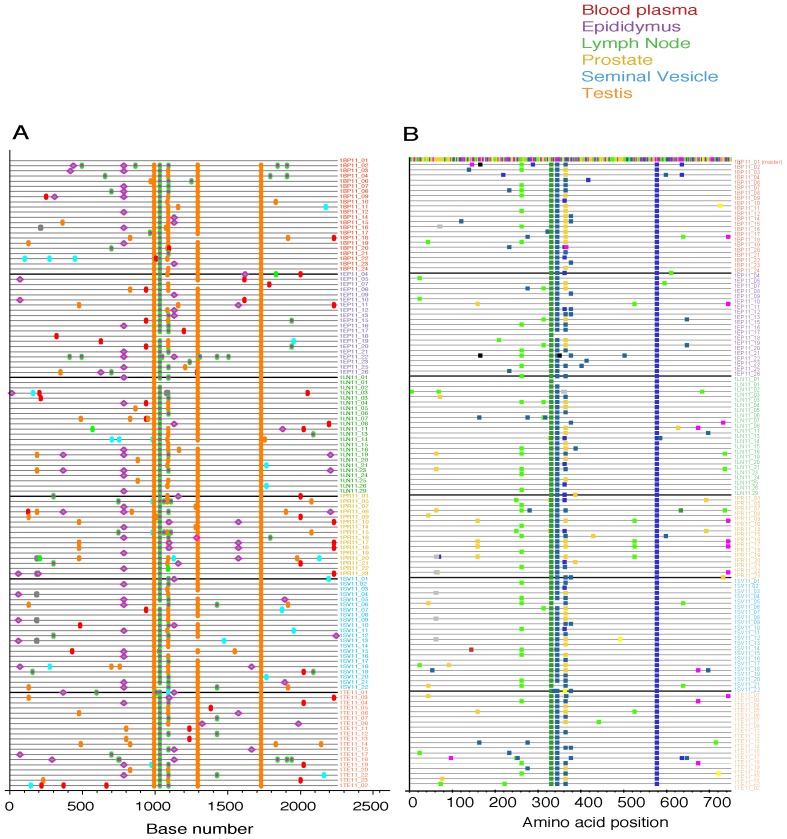
Highlighter plots of SGA derived *env* sequences from macaque 36338. (A) Highlighter plot of SIV *env* nucleotide sequences from blood plasma (red), epididymis (purple), axillary lymph node (green), prostate (yellow), seminal vesicle (blue) and testis (orange) in male macaque 36338, 11 weeks post infection with SIVmac251. Individual nucleotide polymorphisms are indicated by green (adenine), red (thymine), yellow (guanine), or blue (cytosine) ticks and are in comparison to master sequence 1BP11_01. Gaps are indicated in gray. (B) Highlighter plot of SIV env amino acid sequences from tissues.

**Figure 2:**
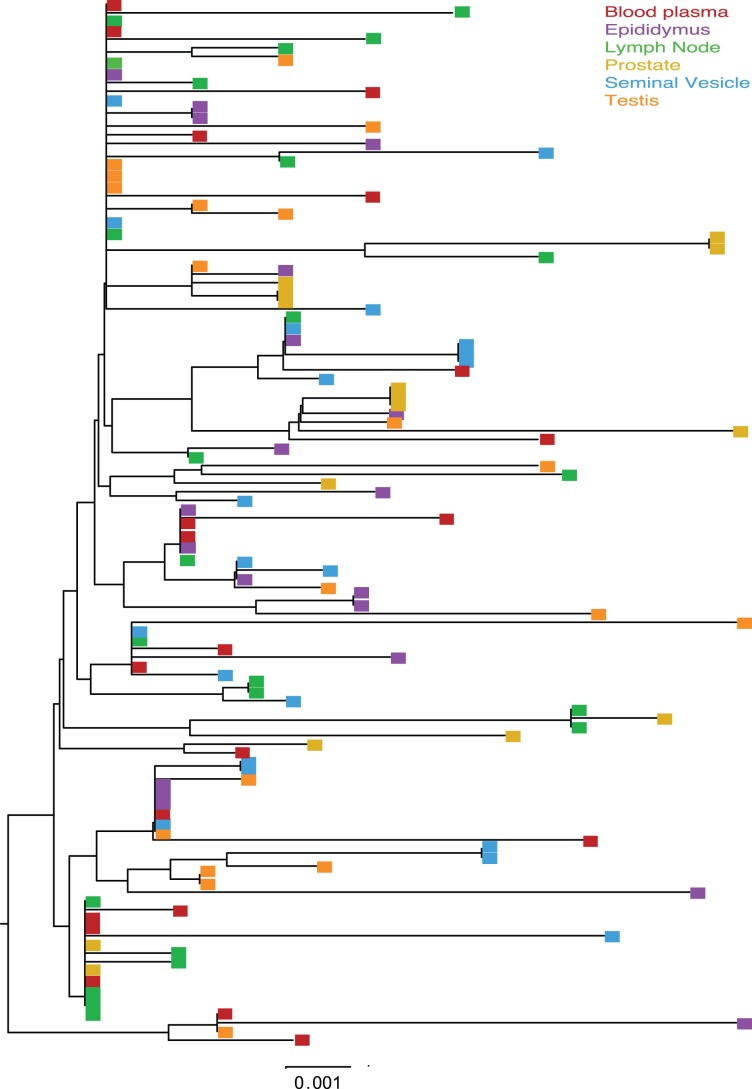
Neighbor Joining tree of SGA derived *env* sequences from macaque 36338. Midpoint rooted Neighbor-joining trees of the *env* sequences from SIVmac251 infected animal 36338 at 11 weeks post infection. Bar represents 0.001 nucleotide substitutions per site.

**Figure 3:**
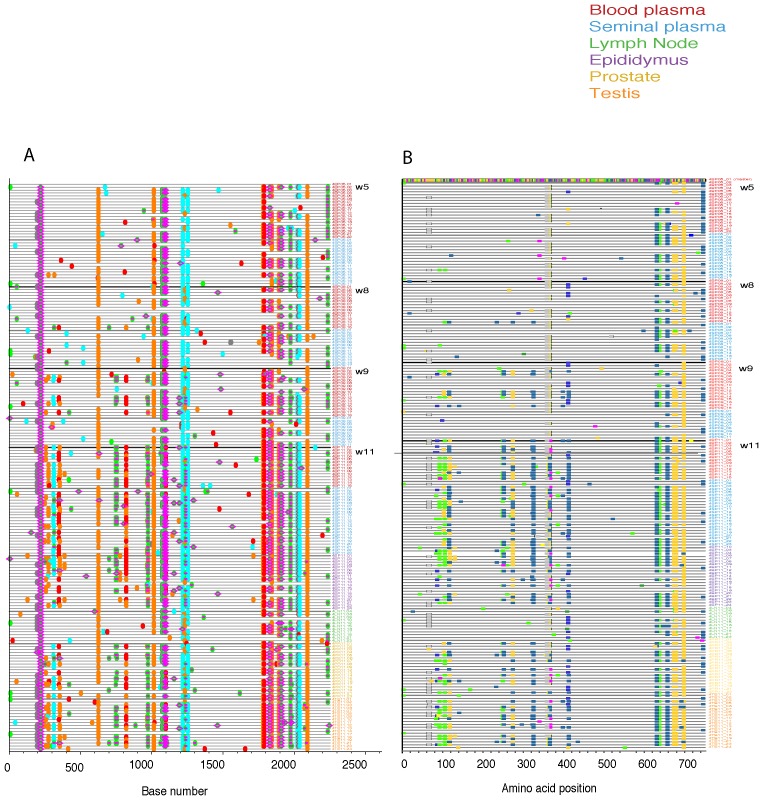
Highlighter plots of SGA derived env sequences from macaque 36199. (A) Highlighter plot of SIV *env* nucleotide sequences from blood plasma (red), epididymis (purple), axillary lymph node (green), prostate (yellow), seminal vesicle (blue) and testis (orange) in male macaque 36199. Sequences from blood plasma and seminal plasma are from 5, 8, 9, and 11 weeks post-infection. Sequences from epididymis, axillary lymph node, prostate, and testis were taken 11 weeks post-infection with SIVmac251. Individual nucleotide polymorphisms are indicated by green (adenine), red (thymine), yellow (guanine), or blue (cytosine) ticks and are in comparison to master sequence 4BP05_01. Gaps are indicated in gray. (B) Highlighter plot of SIV *env* amino acid sequences from tissues.

**Figure 4:**
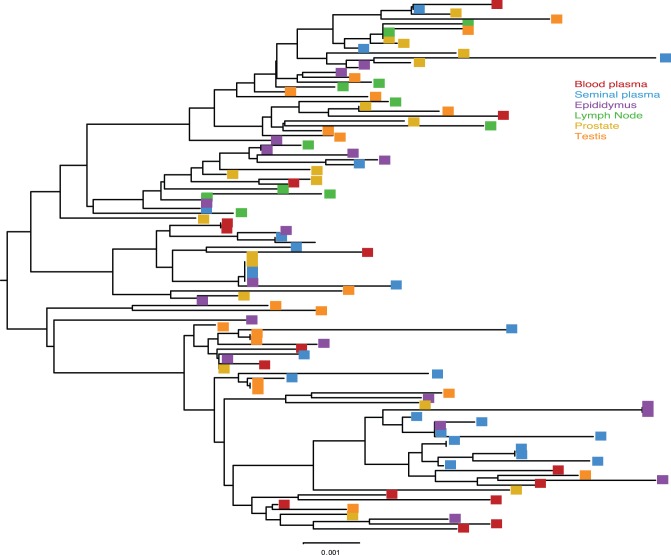
Neighbor joining tree of SGA derived *env* sequences from macaque 36199. Midpoint rooted Neighbor-joining tree of the *env* sequences from SIVmac251 infected animal 36199, 11 weeks post-infection. Bar represents 0.001 nucleotide substitutions per site.

Cross sectional analysis of the SIVmac251 viral populations in blood plasma, systemic lymph node, and genital tissues was performed on two of the macaques (36338 and 36199). In most cases a minimum of 30 SGA derived sequences were examined for each tissue compartment using nucleotide and amino acid Highlighter plots, and Neighbor-Joining tree phylogenic analysis (Fig 1–4).

All tissues collected from animal 36338 had a similar population of SIV *env* variants. The Highlighter plot showed that by the time of necropsy there were a number of common mutations relative to the master sequence that had become fixed in most SIV variant nucleotide (Fig 1A) and amino acid (Fig 1B) sequences. In addition, although unique low frequency variants were present in each tissue, no specific SIV variants were enriched in a particular anatomical site and there was no evidence of signature sequences for variants found in a particular tissue. Finally, visual inspection of the Neighbor-Joining phylograms demonstrated that all variants were found in all tissue compartments although the frequency varied. Thus, the viral populations in the blood of animal 36338 are not distinct from those in the genital tissues (Fig 2).

Visual inspection of the nucleotide (Fig 3A) and amino acid (Fig 3B) Highlighter plots of the *env* variant population in the blood plasma of animal 36199 showed increasing diversity from week 5 to week 11 PI. Several nucleotide polymorphisms became fixed in the blood plasma at week 9 before being detected in seminal plasma, suggesting that these variants arose in the systemic compartment before accessing genital tissues. SIV *env* variants found early in the infection were evenly distributed among all of tissues, and novel *env* variants arising later in infection became successfully infected most, but not all, systemic lymphoid tissues. Interestingly, the axillary lymph node in this animal lacks some of the variants that were present in genital tissues collected at the same time point and appears to largely harbor a collection of SIV*env* variants that arose early in the infection. This unexpected finding suggests that, at least in this animal, not all *env* variants in blood could seed all lymphoid tissues and that the variants that established infection early were able to monopolize the available target cells in some tissues (Fig 4).

## Discussion

Although HIV is shed in semen, the anatomic site of virus entry into the MGT secretions is unknown. We studied SIV localization in the semen and genital tract tissues of viremic male rhesus macaques experimentally infected by penile SIV inoculation. Penile SIV transmission mimics the key virological and epidemiological features of HIV transmission in men [10]. This novel infection route provides a useful model for male human HIV transmission [9]. It was previously reported that in male cynolomologus macaques infected with SIVmac251 by IV inoculation, the vRNA levels in genital tract correlate with the viral load in the blood [8] and our results in rhesus macaques support that conclusion as the vRNA levels in all 4 MGT tissues tested (prostate, seminal vesicle, epididymis, testis) directly correlated with plasma vRNA levels.

The MGT does not seem to be a significant site of SIV replication. In fact, although SIV RNA was found in the axillary lymph nodes of all 5 animals, it was only found in in the reproductive tract tissues of the 3 monkeys with the highest plasma vRNA levels (>10^6^ vRNA copies/ml plasma). Further, the levels of vRNA in all the genital tract tissues were considerably lower than in the lymph node of the same animal. In the 2 animals with lowest plasma vRNA levels (<10^4^ vRNA copies/ml plasma), SIV RNA was undetectable in genital tract tissues. This relationship suggests that the tissues of the male genital tract contain relatively few SIV-infected cells and that genital tract tissues are not major sites of virus replication. Of note in the terminal stages of AIDS, granulomatous inflammation marked by syncytial giant cells can develop on the testes and epididymis of SIV-infected rhesus macaques and many of the cells in this inflammatory condition are SIV-infected [21]. Thus, genital tract inflammation can serve as a major source of SIV in the MGT. Because tissues from only 3 animals were examined, it is not possible to conclude that there is, or is not, a pattern in the relative vRNA levels among genital tissues, although the high levels of vRNA in the testis of 36199 seem unusual.

HIV RNA is readily detected in human seminal plasma and in many studies the vRNA levels and population of viral variants in the seminal plasma reflect the vRNA levels and virus populations in blood plasma [1], [22]–[24]. These results suggest that, as we describe above for rhesus macaques, the blood is the major source of virus in seminal fluid. However, there are reports of discordant levels of vRNA and distinct populations of virus variants in the seminal fluid and blood plasma of some men [1], [4], [25]–[28]. Leading to the conclusion that, in these cases, the genital tract tissues and not the blood is the major source of virus in seminal fluid. Comparison of the SIVmac251 *env* viral populations in blood plasma, systemic lymph node, and genital tract tissues was performed in two macaques. We found a striking similarity in the viral populations in the blood and male genital tract tissues after penile SIV transmission in both of the animals that we examined during the early setpoint phase of infection. The Neighbor-Joining phylograms clearly showed that within one animal, all SIV *env* variants were found in all tissue compartments. While in the second animal there were unique *env* variants in the axillary LN but all variants in the MGT were also in the blood and systemic tissues. Thus, in these 2 animals the populations of SIV*env* variants in the genital tissues were not distinct from those in the systemic tissues. In contrast, it was recently reported that found that although SIV variants were similar in blood and semen during acute infection, by the time plasma vRNA levels are at a set point, compartmentalization of SIV variants was apparent in 4 of 7 monkeys examined [38]. The apparent discrepancy in the results of the 2 studies likely reflects the small number of animals examined in the current study. In fact, the global distribution of variants among anatomic compartments in the 2 animals in the present study is similar to the findings of 3 of 7 animals examined by Whitney et al. [29]. Compartmentalization of SIV variants between the male genital and blood was also reported in a separate study [8], thus it is very possible that evidence of separate virus variant populations in the genital tract and systemic tissues of individual animals could have been found if we had studied more animals. The results reported here and by others [8], [29] suggest that as in men, the extent to which there is compartmentalization between the genital tract and blood varies among the individual macaques.

The relative contribution of cell free HIV in seminal plasma and HIV-infected cells to transmission has been long debated [30]. While HIV nucleic acid can be detected in both fluid and cell fractions of semen [30], seminal plasma is toxic to tissue culture cells, and thus isolating infectious virus from these samples is difficult. Although a few studies in macaques have shown that SIV-infected cells placed into the vagina can transmit infection, either large number of cells had to be used [31] or the vaginal mucosa had to be chemically disrupted prior to inoculation to achieve reliable transmission [32], [33]. In this study, SIV RNA was in most of the semen samples collected and the seminal plasma was vRNA+ much more frequently than the seminal mononuclear cells. Thus to the extent SIV is sexually transmitted in rhesus macaques, seminal plasma is the likely to be the source of the transmitted virus. In conclusion, our findings demonstrate that the vRNA levels and viral variant populations in the blood and genital tract tissues are similar and this is consistent with many human HIV infections. These results confirm the utility of the rhesus macaque as an animal model for studying HIV acquisition and shedding in men.
